# Real-world patient-reported outcomes and physician satisfaction with poly (ADP-ribose) polymerase inhibitors versus chemotherapy in patients with germline *BRCA1/2*-mutated human epidermal growth factor receptor 2–negative advanced breast cancer from the United States, Europe, and Israel

**DOI:** 10.1186/s12885-022-10325-9

**Published:** 2022-12-22

**Authors:** Reshma Mahtani, Alexander Niyazov, Bhakti Arondekar, Katie Lewis, Alex Rider, Lucy Massey, Michael Patrick Lux

**Affiliations:** 1 Miami Cancer Institute, 1228 S Pine Island Road, Plantation, Miami, FL 33324 USA; 2 Pfizer Inc, 235 42nd St, New York, NY 10017 USA; 3 Pfizer Inc, 500 Arcola Road, Collegeville, PA 19426 USA; 4 Adelphi Real World, Adelphi Mill, Cheshire, Bollington, SK10 5JB UK; 5 Kooperatives Brustzentrum Paderborn, Frauenklinik St. Louise, Paderborn, St. Josefs-Krankenhaus, Salzkotten, Frauen- und Kinderklinik St. Louise, Salzkotten Husener Straße 81, 33098 Paderborn, Germany

**Keywords:** Advanced breast cancer, *BRCA1/2* mutation, Chemotherapy, Patient-reported outcomes, Poly (ADP-ribose) polymerase inhibitors, Quality of life, Real-world

## Abstract

**Background:**

In clinical trials, poly (ADP-ribose) polymerase inhibitors (PARPi) versus chemotherapy resulted in significantly improved progression-free survival, manageable adverse event profiles, and favorable patient-reported outcomes (PROs) in patients with human epidermal growth factor receptor 2–negative (HER2−) advanced breast cancer (ABC) and germline *BRCA1/2* mutations (g*BRCA1/2*mut). The objective of this study was to evaluate PROs and physician satisfaction with treatment in patients with g*BRCA1/2*mut HER2− ABC receiving PARPi or physician’s choice of chemotherapy in a multi-country, real-world setting.

**Methods:**

This retrospective analysis used data from the Adelphi Real World ABC Disease Specific Programmes in the United States, European Union, and Israel. PROs were assessed at a single timepoint using the EuroQol 5-Dimensions 5-Level (EQ-5D-5L) scale, Cancer Therapy Satisfaction Questionnaire (CTSQ), and European Organization for Research and Treatment of Cancer Quality-of-Life Questionnaire Core 30 (EORTC QLQ-C30) and the breast cancer−specific module (QLQ-BR23). Baseline PROs were not assessed. Physician satisfaction with treatment scores was dichotomized to a 0/1 variable (0 = very dissatisfied/dissatisfied/moderately satisfied; 1 = satisfied/very satisfied). Scores were compared using inverse-probability−weighted regression adjustment, controlling for multiple confounding factors.

**Results:**

The study included 96 patients (PARPi, *n* = 38; platinum/non–platinum-based chemotherapy, *n* = 58). Patients receiving PARPi versus chemotherapy reported significantly better scores on the EQ-5D-5L Health Utility Index. On the EORTC QLQ-C30 functional scales, patients receiving PARPi reported significantly better scores (mean ± SE) for physical functioning (80.0 ± 2.4 vs 71.9 ± 3.4; *p* < 0.05) and social functioning (82.0 ± 6.2 vs 63.6 ± 3.7; *p* < 0.05) and, on the symptom scales, reported significantly better scores for constipation (1.9 ± 1.8 vs 18.7 ± 3.2; *p* < 0.001), breast symptoms (0.4 ± 3.9 vs 13.3 ± 2.6; *p* < 0.01), arm symptoms (2.6 ± 1.3 vs 11.4 ± 2.4; *p* = 0.001), and systemic therapy side effects (13.5 ± 1.8 vs 29.4 ± 2.3; *p* < 0.001). In contrast, patients receiving chemotherapy scored significantly better on the nausea/vomiting scale (18.3 ± 2.8 vs 34.5 ± 5.1; *p* < 0.01). Patients receiving PARPi reported numerically better satisfaction scores on the CTSQ scales. Physicians were more likely to be satisfied/very satisfied with PARPi versus chemotherapy (95.4% ± 7.3% vs 40.8% ± 6.2%; *p* < 0.001).

**Conclusions:**

The PRO findings in this real-world population of patients with g*BRCA1/*2mut HER2− ABC complement those from the pivotal clinical trials, providing further support for treatment with PARPi in these patients.

**Supplementary Information:**

The online version contains supplementary material available at 10.1186/s12885-022-10325-9.

## Key summary points

- In clinical trials, poly (ADP-ribose) polymerase inhibitors (PARPi) versus chemotherapy resulted in significantly improved progression-free survival and favorable patient-reported outcomes in patients with human epidermal growth factor receptor 2–negative (HER2−) advanced breast cancer (ABC) and germ-line *BRCA1/2* mutations (g*BRCA1/2*mut).
- Patient-reported outcomes in patients with g*BRCA1/2*mut HER2− ABC receiving PARPi or physician’s choice of chemotherapy in real-world clinical practice are unknown.
- Patients receiving PARPi versus chemotherapy reported a significantly improved or similar quality of life using the European Quality-of-Life 5-Dimensions 5-Levels and European Organization for Research and Treatment of Cancer QLQ-C30 instruments.
- Physicians were more likely to be satisfied/very satisfied with PARPi versus chemotherapy.
- Patient-reported outcomes in this real-world population of patients with g*BRCA1/*2mut HER2− ABC complement those from pivotal clinical trials, providing further support for treatment with PARPi in these patients.

## Background

Poly (ADP-ribose) polymerase inhibitors (PARPi) olaparib and talazoparib have been approved by the US Food and Drug Administration and European Medicines Agency and in Israel for the treatment of patients with human epidermal growth factor receptor 2–negative (HER2−) locally advanced and/or metastatic breast cancer harboring a germline mutation in the breast cancer susceptibility gene 1 or 2 (g*BRCA1/2*mut) [1, 2]. The approvals were based primarily on findings from the OlympiAD and EMBRACA randomized open-label trials, which demonstrated significantly improved progression-free survival (PFS) and a manageable adverse event profile in patients with *gBRCA1/2*mut HER2− advanced breast cancer (ABC) who received olaparib or talazoparib compared with patients who received physician’s choice of chemotherapy (OlympiAD: olaparib versus capecitabine, vinorelbine, or eribulin; EMBRACA: talazoparib versus capecitabine, vinorelbine, eribulin, or gemcitabine) [3, 4]. In the OlympiAD trial, median PFS was significantly longer in the olaparib group compared with the chemotherapy group (7.0 vs 4.2 months; hazard ratio for disease progression or death, 0.58; *p* < 0.0001) [3]. Similarly, in the EMBRACA trial, median PFS was significantly longer in the talazoparib group compared with the chemotherapy group (8.6 vs 5.6 months; hazard ratio for disease progression or death, 0.54; *p* < 0.001) [4]. In OlympiAD and EMBRACA, no statistically significant overall survival benefit was observed by olaparib or talazoparib, respectively, compared with physician’s choice of chemotherapy, [5, 6]. These PARPi are now available in multiple countries for the treatment of g*BRCA1/2*mut HER2− ABC [7].

Extending patients’ lives is an important goal of treatment, but, in ABC, treatment is palliative, and one of the most important goals is the maintenance or improvement of quality of life (QoL) [8]. Guidelines recommend that, in addition to efficacy and safety, the effect of treatment on patients’ QoL should be considered [7]. In the OlympiAD and EMBRACA trials, olaparib and talazoparib, respectively, demonstrated significantly favorable patient-reported outcomes (PROs) compared with physician’s choice of chemotherapy [3, 4]. However, there is little information on the effect of PARPi on QoL in patients with HER2− ABC in real-world clinical practice. Using real-world evidence to complement findings from clinical trials is important because clinical trials can exclude a substantial portion of the population (eg, elderly patients, patients with comorbidities, or patients with different prior therapies who are not reflected in clinical trials), making it difficult to generalize findings to larger, more inclusive patient populations [9]. In this study, we compare real-world PROs among patients with g*BRCA1/2*mut HER2− ABC receiving PARPi or chemotherapy (platinum- and non–platinum-based therapy and chemotherapy in combination with immunotherapy) in Germany, France, Italy, Spain, the United States, and Israel.

## Methods

### Data source and study design

Data were obtained from the Adelphi Real World Advanced Breast Cancer Disease Specific Programmes™ (DSP; Bollington, UK) conducted from October 2019 through March 2020 in the United States, France, Germany, Italy, Spain, and Israel. Disease Specific Programmes are large, multinational, point-in-time surveys of physicians and their patients presenting in a real-world clinical setting that assess disease management, disease burden, and associated treatment effects [10].

**Fig. 1:**
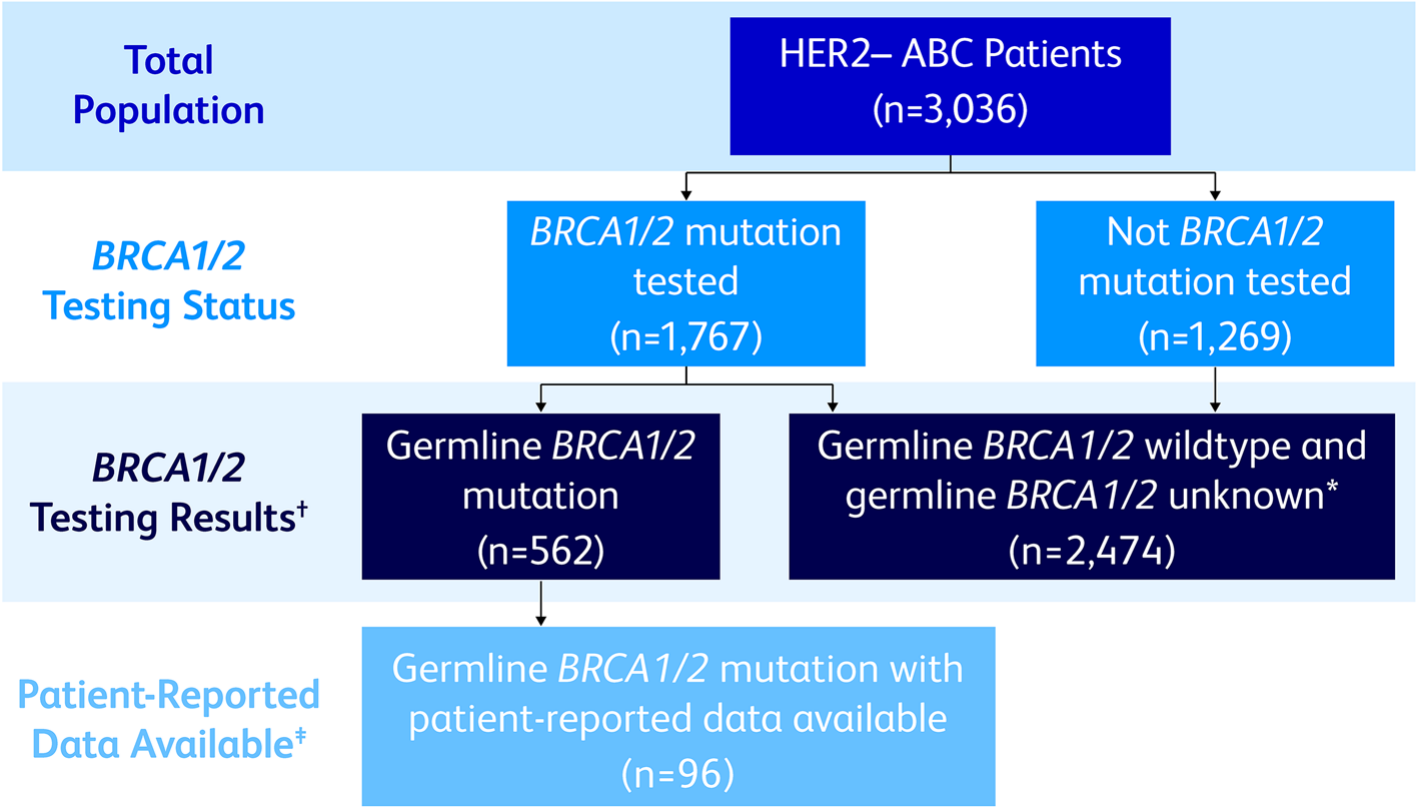
*BRCA1/2*-Mutation Status Testing. ABC, advanced breast cancer; *BRCA1/2*, breast cancer susceptibility gene 1 or 2; HER2–, human epidermal growth factor receptor 2–negative; IPWRA, inverse-probability–weighted regression adjustment. *Includes: not tested; not known to have a *BRCA1/2* germline mutation test result; not known to have *BRCA1/2* germline and somatic wildtype test results. ^**†**^Of the total population of 3036 patients, 1239 agreed to participate in the patient-reported outcomes analysis. ^‡^Patients had all patient-reported outcome and covariate data available for the IPWRA analysis

Medical oncologists treating ≥5 patients with ABC per month and actively involved in patient prescribing decisions were recruited by local fieldwork teams. Physicians provided patient record forms for 8 eligible patients: 4 patients receiving first-line advanced treatment and 4 patients receiving second- or later-line advanced treatment. Eligible patients were aged ≥18 years with stage IIIb or IV HER2− breast cancer, receiving therapy for ABC at the time of data collection, and not currently participating in a clinical trial. Physicians were invited to complete 1 to 4 patient record forms (PRFs) regarding patients with known somatic or germline *BRCA1/2*muts who fit the overall eligibility criteria. Only patients with known g*BRCA1/2*muts were included in this analysis. Verification that *BRCA1/2*mut testing was done on blood, saliva, and/or buccal samples was used to confirm that physicians were abstracting data from patients with g*BRCA1/2*muts as opposed to somatic *BRCA1/2* mutations in the tumor. For US-based patients, the test type (somatic or germline) was confirmed by the laboratory where the testing was performed; data for laboratory confirmation of test type was not available for the 4 EU countries or Israel (Fig. 1).

The PRF included detailed questions on patient demographics, clinical assessments, clinical outcomes, adverse events experienced at the time of data collection among treated patients, treatment history, and physician-rated satisfaction with treatment. The treating physicians completed the data collection form using patient medical records as well as their clinical judgement and diagnostic skills consistent with their decision-making process during routine clinical practice. Each patient with a PRF was also invited to complete an optional written patient form independently of their physician immediately after consultation. The patient form contained questions on their education, employment status, input in treatment decisions, and current disease status. Patients were also asked to complete several PRO questionnaires that assessed their health-related QoL. PRO questionnaires were collected at 1 time point, the time of data collection, which corresponds to the treatment duration. Baseline PRO questionnaires were not collected.

Patients provided informed consent for use of their anonymized and aggregated data for research and in scientific publications. Data were aggregated and de-identified before receipt by Adelphi Real World. This research was approved by the Western Institutional Review Board (study protocol AG8643). Data collection was undertaken in line with European Pharmaceutical Marketing Research Association guidelines [11] and, as such, did not require ethics committee approval. This study was administered in full accordance with relevant legislation at the time of data collection, including the US Health Insurance Portability and Accountability Act of 1996 [12].

### PRO assessments

Physicians invited patients to complete the following validated PRO questionnaires: the European Quality of Life 5-Dimensions 5-Levels (EQ-5D-5L), a widely used generic instrument that describes health-related QoL [13]; the European Organization for Research and Treatment of Cancer Quality-of-Life Questionnaire Core 30 (EORTC QLQ-C30), an integrated system for assessing health-related QoL in cancer patients [14, 15]; the EORTC breast cancer–specific module QLQ-BR23 [16]; and the Cancer Therapy Satisfaction Questionnaire (CTSQ) [17], which measures patient satisfaction with their current therapy.

The EQ-5D-5L comprises 5 dimensions, each being scored as numeric variables of 1 to 5 (1 = no problem; 5 = extreme problem) [13]. EQ-5D-5L summary index values derived from the EQ-5D-5L dimensions were also determined. The EQ-5D-5L was scored (Van Hout crosswalk method) based on standard individual 3-level value sets from the corresponding individual countries, except for Israel where the United Kingdom value sets were used [18]. The EQ-5D-5L includes a visual analog scale (VAS) that provides a quantitative measure of the patient’s perception of their overall current health, and for which the endpoints are “the best health you can imagine” and “the worst health you can imagine.”

The EORTC QLQ-C30 is composed of multi-item scales and single-item measures, including 5 functional scales, 3 symptom scales, a global health status (GHS)/QoL scale, and 6 single items [15]. Each multi-item scale includes a different set of items (ie, no item occurs in more than one scale). Items are scored on a 4-point scale from 1 (not at all) to 4 (very much), except for items contributing to the GHS/QoL, which are scored on a 7-point scale. All of the scales and single-item measures range in score from 0 to 100, with a high scale score representing a higher response level. The breast cancer module (QLQ-BR23) incorporates 5 multi-item scales that assess systemic therapy side effects, arm symptoms, breast symptoms, body image, and sexual functioning and single items to assess sexual enjoyment, hair loss, and future perspective [15]. The scoring for the QLQ-BR23 is the same in principle as that for the function and symptom scales/single items of the QLQ-C30.

The CTSQ contains 16 items and assesses 3 domains: Expectations of Therapy, Feelings About Side Effects, and Satisfaction With Therapy [17]. Items are scored on a scale of 1 to 5, with 1 representing the worst response and 5 the best response.

### Physician satisfaction

Physicians were asked to describe their satisfaction with the current treatment for their patient’s ABC (very dissatisfied to very satisfied). The scores were dichotomized to a 0/1 variable (0 = very dissatisfied/dissatisfied/moderately satisfied; 1 = satisfied/very satisfied).

### Statistical analysis

Descriptive summary statistics, including means, were calculated for continuous variables. Frequency counts and percentages were calculated for categorical variables. For any missing values, patients were removed from all analyses in which that variable was used. There was no imputation of missing data.

Patient demographics and baseline clinical characteristics were balanced using inverse-probability–weighted regression adjustment (IPWRA). The covariates balanced within the IPWRA were age at current treatment initiation, Charlson Comorbidity Index, baseline toxicities (constitutional, pulmonary, gastrointestinal, dermatologic, neurologic, psychological, physical, and pain), hormone receptor status, Eastern Cooperative Oncology Group (ECOG) performance status at current treatment initiation, stage at current treatment initiation, and number of treatments received in the advanced setting. Unadjusted demographics/clinical characteristics were analyzed using Student’s *t* tests and Fisher exact tests.

The IPWRA-adjusted CTSQ Patient Satisfaction, EQ-5D-5L Health Utility dimensions, EORTC QLQ-C30 and QLQ-BR23 scores, and physician satisfaction scores were compared between platinum/non–platinum-based chemotherapy (± immunotherapy) and PARPi monotherapy by logistic regression analysis. Mean scores ± standard error and 95% confidence intervals are presented. *P* values < 0.05 were considered significant. Analyses were performed using Stata v16.1 or later (StataCorp, College Station, TX, USA; 2019).

## Results

### Patient characteristics

Of the 562 patients identified with g*BRCA1/2*mut, 96 female patients had all patient-reported data available and were included in the study (Fig. 1). The remaining 466 patients had either missing PRO or demographic and clinical characteristic covariate data (ie, data not provided by physicians). Statistically significant differences in age, hormone receptor status, ECOG performance status, gastrointestinal toxicity, blood/circulatory toxicity, and physical toxicity between the 96 patients included in the study and the remaining 466 patients were identified (see Additional file 1). Of the 96 patients included in the study, 38 (39.6%) were receiving a PARPi and 58 (60.4%) were receiving platinum/non–platinum-based chemotherapy (± immunotherapy) at the time of data collection. Among patients receiving chemotherapy, 29 (50.0%) were receiving platinum-based and the remaining 29 (50.0%) were receiving non–platinum-based chemotherapy. The type of chemotherapy treatments that patients received are shown in Table 1. Carboplatin was the most common chemotherapy, received by 39.7% of the chemotherapy-treated patients. Capecitabine, the only oral therapy, was received by 13.8% of chemotherapy-treated patients. The unadjusted mean ± SD (range) duration of treatment, which corresponds with the time patients were on treatment when they reported PROs, was similar in the PARPi and chemotherapy groups, 123.7 ± 86.3 (4.0–321.0) days and 118.6 ± 123.8 (0–868.0) days (*p* = 0.82), respectively. After IPWRA adjustment, the mean duration of treatment for patients receiving PARPi or chemotherapy remained similar: 101.4 and 105.6 days (*p* = 0.86), respectively.

**Table 1:** Types of chemotherapies received by patients treated with chemotherapy

Current Therapy, n (%)	Chemotherapy Patients (***n*** = 58)
Carboplatin	23 (39.7)
Paclitaxel	17 (29.3)
Cyclophosphamide	11 (19.0)
Capecitabine	8 (13.8)
Nab-Paclitaxel	7 (12.1)
Cisplatin	6 (10.3)
Vinorelbine	6 (10.3)
Gemcitabine	6 (10.3)
Doxorubicin Liposomal	5 (8.6)
Doxorubicin	4 (6.9)
Epirubicin	4 (6.9)
Eribulin	3 (5.2)
Docetaxel	1 (1.7)

Significant differences at baseline in the Charlson Comorbidities Index; dermatologic, neurologic, psychological, and physical toxicities; cancer stage at treatment initiation; and line of treatment between patients receiving PARPi or chemotherapy were observed (Table 2). After IPWRA adjustment, demographic and clinical characteristics were well balanced (Table 2).

**Table 2:** Demographic and clinical characteristics by treatment received among patients with g*BRCA1/2*mut HER2− ABC

	Unadjusted			IPWRA-Adjusted	
	PARPi Monotherapy (*n* = 38)	Chemotherapy^a^ (*n* = 58)	*P* Values	PARPi Monotherapy (*n* = 38)	Chemotherapy^a^ (*n* = 58)
Mean age at therapy initiation, years	50.7	51.3	0.829^†^	50.2	50.5
Female sex, %	100	100	1.000^‡^	100	100
White race, %	86.8	93.1	0.476^‡^	91.9	85.8
Premenopausal, %	44.7	41.4	0.834^‡^	43.7	43.7
HR+/HER2− status, %	31.6	36.2	0.667^‡^	34.5	38.4
Currently known to be working full/part time, %	28.9	24.1	0.640^‡^	30.4	25.2
Charlson Comorbidities Index, mean	6.2	7.5	**0.017**^†^	7.1	7.1
Baseline toxicities,^b^ %					
Constitutional	47.4	36.2	0.296 ^‡^	29.6	33.9
Pulmonary	31.6	19.0	0.221 ^‡^	20.1	19.3
Gastrointestinal	26.3	15.5	0.204 ^‡^	14.7	15.4
Dermatologic	36.8	8.6	**0.001** ^‡^	19.2	15.7
Neurologic	18.4	1.7	**0.006** ^‡^	8.5	6.1
Psychological	18.4	5.2	**0.047** ^‡^	9.9	9.4
Physical	63.2	29.3	**0.002** ^‡^	34.4	38.8
Pain	34.2	20.7	0.159 ^‡^	23.4	23.8
ECOG performance status at treatment initiation, mean	0.7	0.8	0.460 ^†^	0.75	0.80
Stage IV at current treatment initiation, %	65.8	89.7	**0.008** ^‡^	82.3	83.0
Line of current treatment, mean	1.9	1.4	**0.002**^†^	1.6	1.6

*ABC* Advanced breast cancer, g*BRCA1/2*mut Germline breast cancer susceptibility gene 1 or 2 mutation, *ECOG* Eastern Cooperative Oncology Group, *HER2–* Human epidermal growth factor receptor 2–negative, *HR+* Hormone receptor–positive, *IPWRA* Inverse-probability–weighted regression adjustment, *PARPi* Poly (ADP-ribose) polymerase inhibitors

^†^ Student’s *t* test

^‡^Fisher’s exact test

^a^Includes platinum- and non–platinum-based regimens

^b^Initial toxicities patients experienced that were retrospectively reported by physicians

Bold *P*-values represent those that are statistically significant (*p* < 0.05)

### PRO health status and patient treatment satisfaction

**Fig. 2:**
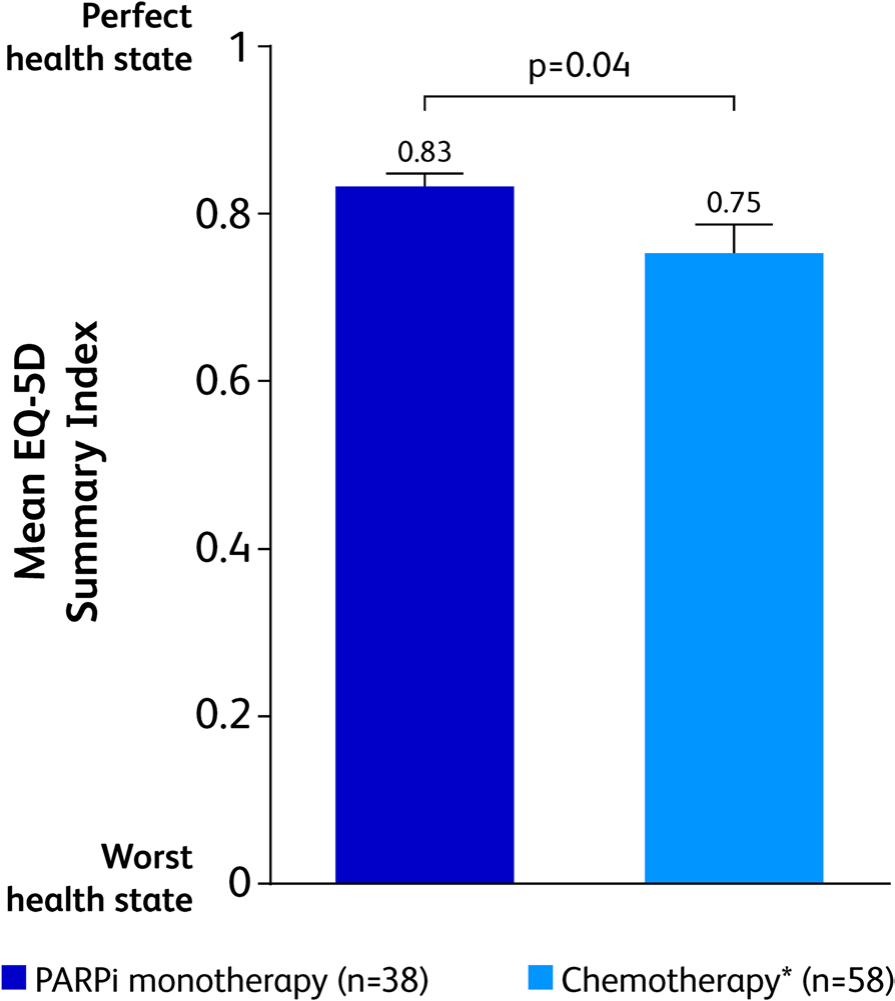
IPWRA Analysis for EQ-5D Health Utility. Mean EQ-5D Summary Index. EQ-5D, EuroQoL-5 Dimension; IPWRA, inverse-probability–weighted regression adjustment; PARPi, poly (ADP-ribose) polymerase inhibitors. Bold *P* values represent those that are statistically significant (*p* < 0.05). *Includes platinum- and non–platinum-based regimens. Error bars represent standard error

Compared with patients treated with chemotherapy, patients treated with PARPi had significantly better EQ-5D Summary Index scores (mean ± SE [95% CI], 0.83 ± 0.02 [0.79–0.87] versus 0.75 ± 0.04 [0.67–0.82]; *p* = 0.04; Fig. 2). The EQ-5D VAS scores for patients treated with chemotherapy and PARPi were similar: 64.9 ± 2.1 and 65.4 ± 4.1 (*p* = 0.92), respectively.

**Fig. 3:**
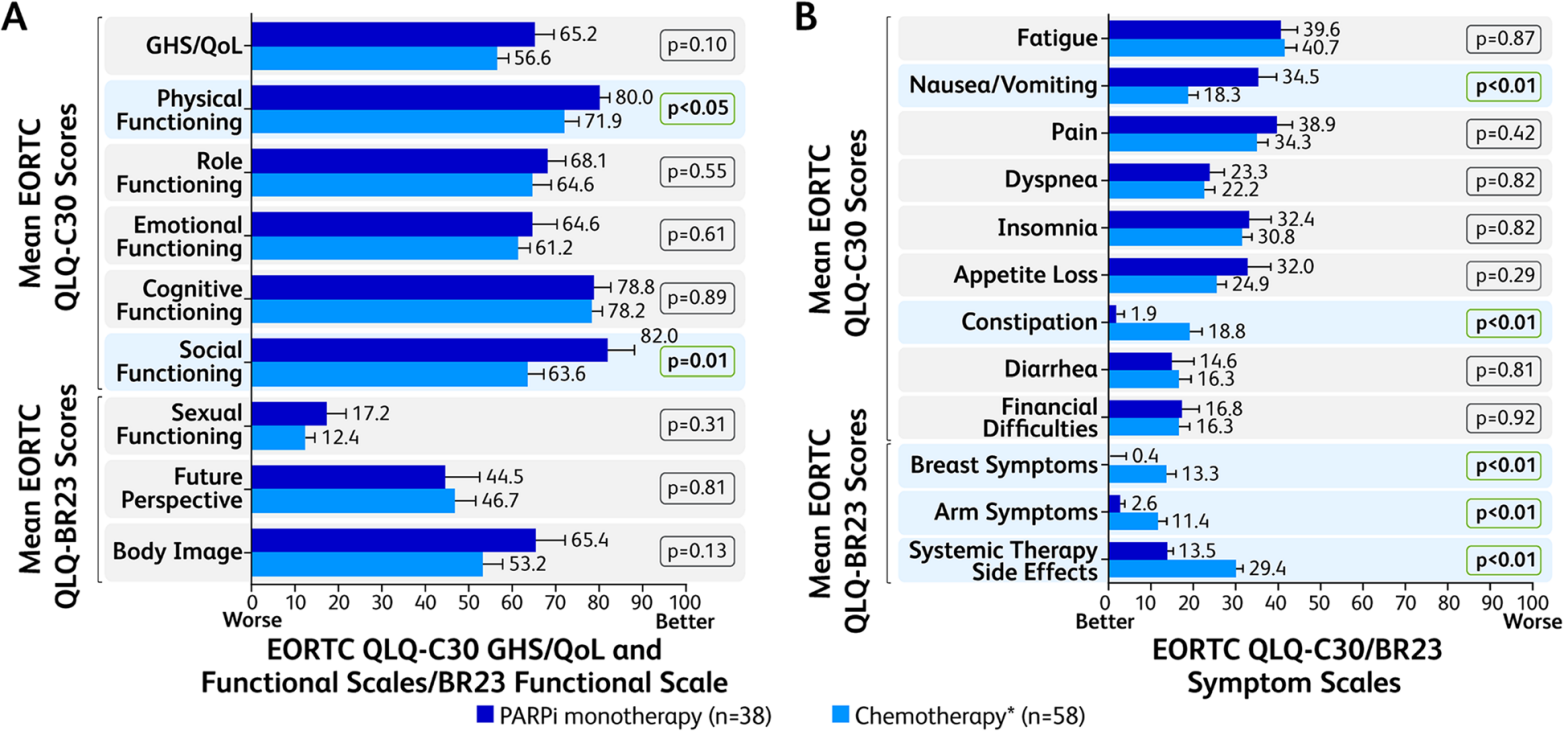
IPWRA Analysis for the EORTC QLQ-C30 and EORTC QLQ-BR23^a^ GHS/QoL, Functional Scales, and Symptom Scales. Mean GHS/QoL and functional scales (**A**) and symptoms scale (**B**) scores adjusted for patient demographic and clinical variables by IPWRA for patients treated with PARPi and chemotherapy. EORTC QLQ-C30, European Organisation for Research and Treatment of Cancer Quality of Life Core 30; EORTC QLQ-BR23, European Organisation for Research and Treatment of Cancer Quality of Life Breast Cancer module; GHS/QoL, global health status/quality of life; IPWRA, inverse-probability–weighted regression adjustment; PARPi, poly (ADP-ribose) polymerase inhibitors. EORTC QLQ-BR23 category Sexual Enjoyment was excluded owing to low sample size. *Includes platinum- and non–platinum-based regimens. Bold *P* values represent those that are statistically significant (*p* < 0.05). Error bars represent standard error

The mean EORTC QLQ-C30 GHS/QoL score was numerically higher in the PARPi-treated patients than those receiving chemotherapy (65.2 ± 4.4 versus 56.6 ± 2.6, *p* = 0.10; Fig. 3A), indicating better QoL for those patients. On the QLQ-C30 functional scales, patients treated with PARPi had a significantly better score compared with patients treated with chemotherapy on the physical functioning scale (80.0 ± 2.4 [75.2–84.8] vs 71.9 ± 3.4 [65.2–78.6]; *p* < 0.05) and social functioning scale (82.0 ± 6.2 [69.9–94.0] versus 63.6 ± 3.7 [56.4–70.8]; *p* = 0.01; Fig. 3A). On the QLQ-C30 symptoms scales, patients treated with PARPi had a significantly better score compared with patients treated with chemotherapy on the constipation scale (1.9 ± 1.8 [− 1.6 to 5.3] vs 18.7 ± 3.2 [12.4 to 25.1]; *p* < 0.01); however, the patients treated with PARPi had a significantly higher (worse) score on the nausea/vomiting scale (34.5 ± 5.1 [24.4–44.6] vs 18.3 ± 2.8 [12.8–23.8]; *p* < 0.01; Fig. 3B**)**. For the breast cancer–specific symptoms scales, patients treated with PARPi had a significantly better score compared with patients treated with chemotherapy on the breast symptoms scale (0.4 ± 3.9 [−7.2 to 7.9] vs 13.3 ± 2.6 [8.2 to 18.4]; *p* < 0.01), arm symptoms scale (2.6 ± 1.3 [0.1–5.1] vs 11.4 ± 2.4 [6.8–16.0]; *p* < 0.01), and systemic therapy side effects scale (13.5 ± 1.8 [1.0–17.0] vs 29.4 ± 2.3 [24.9–33.9]; *p* < 0.01; Fig. 3B).

**Fig. 4:**
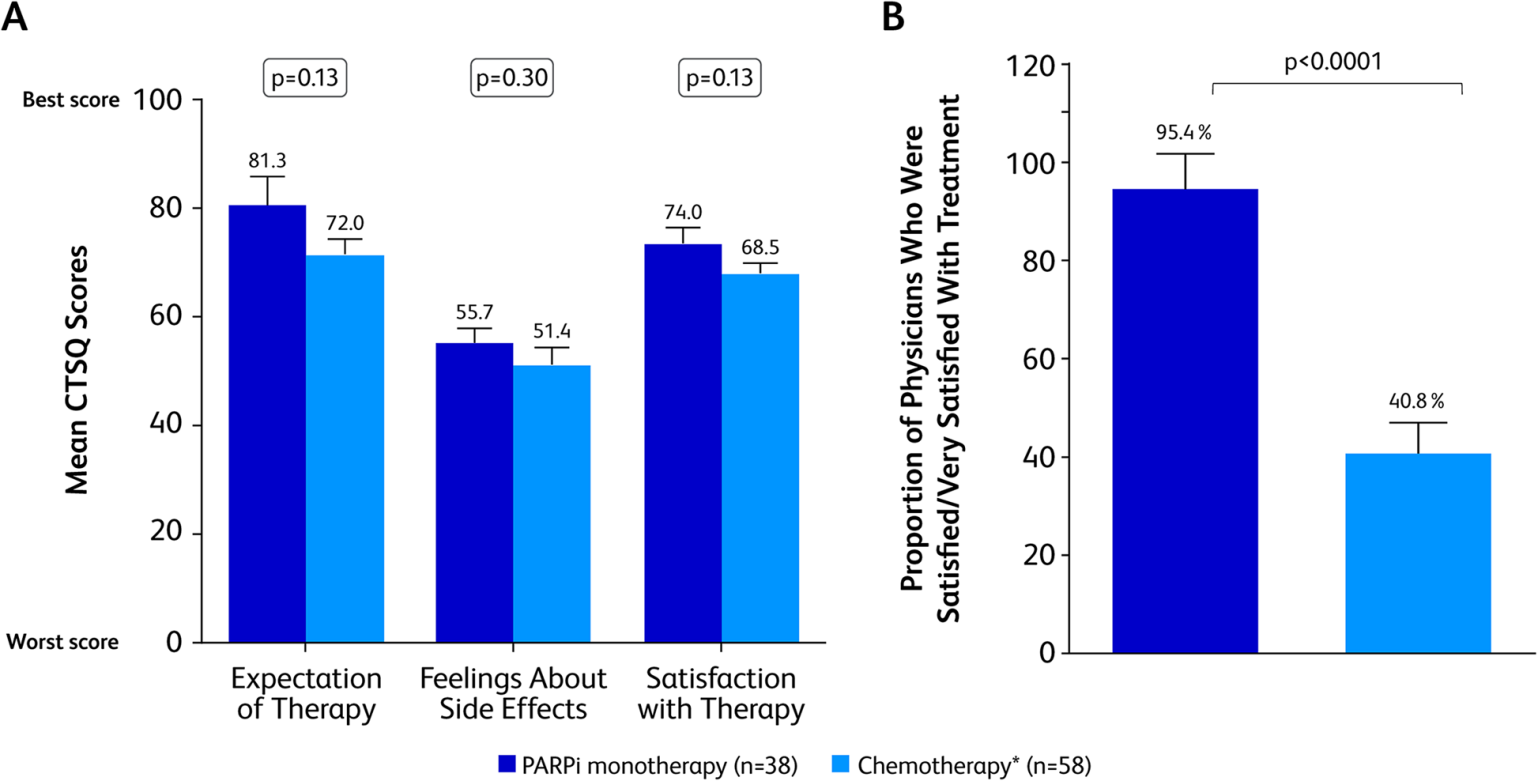
IPWRA Analysis for CTSQ Scores and Physician Satisfaction with Treatment. Mean scores for 3 CTSQ domains (**A**) and proportion of physicians satisfied with treatment (**B**) adjusted for patient demographic and clinical variables by IPWRA for patients treated with PARPi or chemotherapy. CTSQ, Cancer Therapy Satisfaction Questionnaire; IPWRA, inverse-probability–weighted regression adjustment; PARPi, poly (ADP-ribose) polymerase inhibitors. *Includes platinum- and non–platinum-based regimens. Error bars represent standard error

Patient experience and satisfaction with PARPi and chemotherapy treatments were evaluated with the CTSQ. Patients receiving PARPi therapy reported numerically better mean scores than patients receiving chemotherapy in the 3 domains of the CTSQ: expectations of therapy (81.3 ± 5.3 vs 72.0 ± 2.8; *p* = 0.13), feelings about side effects (55.7 ± 2.7 vs 51.4 ± 3.2; *p* = 0.30), and satisfaction with therapy (74.0 ± 3.0 vs 68.5 ± 1.9; p = 0.13; Fig. 4A).

### Physician treatment satisfaction

Physicians were asked to describe their satisfaction with the current treatment for their patients. After adjusting for confounding factors using IPWRA, physicians were significantly more likely to be satisfied/very satisfied with PARPi versus chemotherapy (mean ± SE, 95.4% ± 7.3% versus 40.8% ± 6.2%, *p* < 0.001; Fig. 4B).

## Discussion

When developing new therapies for patients with ABC, delaying progression of disease while maintaining health-related QoL is an important goal [8]. PARPi have recently been approved to treat patients with g*BRCA1/2*mut HER2− ABC. Clinical studies have evaluated QoL measures in patients with g*BRCA1/2*mut HER2− ABC receiving PARPi; however, there is little information on QoL outcomes for patients being treated in routine clinical practice. In this study, 4 different PRO instruments were used, each showing better QoL scores on multiple functional and symptom indices for patients being treated with PARPi compared with patients being treated with physician’s choice of chemotherapy in a multinational real-world population without the strict inclusion and exclusion criteria of clinical studies, which often exclude a substantial portion of the population [9].

In the OlympiAD and the EMBRACA trials, PROs for both olaparib and talazoparib, respectively, were evaluated longitudinally using the EORTC-QLQ-C30, and also with the QLQ-BR23 breast cancer module for talazoparib [3, 4]. Significant overall improvements and significant delays in the time to clinically meaningful deterioration in the GHS/QoL were reported for both the olaparib and talazoparib cohorts compared with standard chemotherapy (non-platinum agents, capecitabine, vinorelbine, eribulin, or gemcitabine) cohorts. Significant improvement in QLQ-BR23 breast symptoms scores and significant delay in time to clinically meaningful deterioration on the breast symptom scale for talazoparib compared with standard chemotherapy were also reported in the EMBRACA trial.

In this cross-sectional analysis, we compared PROs among patients receiving PARPi (olaparib or talazoparib) or chemotherapy, although, unlike in the OlympiAD and the EMBRACA trials, the chemotherapy cohort in this study included many patients (50%) who received platinum-based chemotherapy. In addition, we note that there were significant differences in baseline comorbidities and toxicities between the PARPi and chemotherapy groups; however, these clinical characteristics were controlled for in the analysis to mitigate their potential effects on the PROs. We observed that the EORTC QLQ-C30 GHS was numerically better for patients treated with PARPi compared with those treated with chemotherapy (65.2 versus 56.6; *p* = 0.10), although the difference did not reach statistical significance. However, we did observe significantly better health status for PARPi therapy versus chemotherapy on the EORTC QLQ-C30 physical and social functioning scales and the constipation scale, as well as the EORTC QLQ-BR23 breast symptoms, arm symptoms, and systemic therapy side effects scales, findings that are consistent with the clinical trials that demonstrate improved PROs with PARPi therapy versus chemotherapy.

Among the largest PRO improvements for talazoparib over chemotherapy on the EORTC QLQ-C30 individual functional scales in the EMBRACA trial were role functioning, physical functioning, and social functioning [19]. In the OlympiAD trial, emotional functioning and social functioning were the 2 most improved functional scales in patients treated with olaparib versus chemotherapy, with the physical functioning scale also showing significant improvement with olaparib [20]. Similarly, in our real-world population, we found social functioning and physical functioning to be the only functional scales that demonstrated significantly better scores for PARPi therapy over chemotherapy, suggesting that physical and social functioning may be particularly relevant QoL indices improved by PARPi. These 2 dimensions of QoL are among the most important in the metastatic setting because patients want to remain mobile, take care of their families, work if necessary, meet friends, and pursue hobbies [21].

On the EORTC QLQ-C30 assessment, we did identify 1 scale, nausea/vomiting, in which mean scores were significantly better for the chemotherapy group than for the PARPi therapy group. This was despite the fact that half the patients in the chemotherapy cohort received platinum-based therapy, which can be nauseating. In the EMBRACA trial, EORTC QLQ-C30 nausea/vomiting symptom scale scores were not significantly different between the talazoparib and the standard chemotherapy treatment groups; however, in the OlympiAD trial, the nausea/vomiting symptom scale score was significantly worse in the olaparib-treated group compared with the standard chemotherapy group. These data suggest that in clinical practice, a focus should be placed on optimal supportive therapy regarding nausea and vomiting in patients receiving PARPi. In addition to providing the patient with detailed information, the prescription of prophylactic medication may be considered to help maintain patient compliance.

In the current study, the CTSQ was also used to evaluate patient experiences and satisfaction with therapy, properties that are not captured by the EORTC QLQ-C30. Although not significant, better scores on the expectations of therapy, feelings about therapy, and satisfaction with therapy scales were reported by patients receiving PARPi therapy compared with those receiving chemotherapy, indicating that patients were at least as satisfied with PARPi as with chemotherapy.

Health-related QoL was also evaluated in the current study with the EQ-5D-5L. We observed that the mean EQ-5D Summary Index (health state) score was significantly better (by 0.08) in the PARPi therapy group compared with the chemotherapy group. Using an anchor-based approach, with the Functional Assessment of Cancer Therapy questionnaire scores as an anchor, Pickard et al. estimated the minimally important differences in the EQ-5D index−based health utility score in cancer patients to be 0.09 in the United Kingdom and 0.06 in the United States [22]. The patient population in the current study included US as well as European and Israeli patients, making it difficult to directly compare the EQ-5D Summary Index reported here with those reported by Pickard et al., although the better EQ-5D Summary Index score for PARPi versus chemotherapy of 0.08 reported here is within, if not exceeds, the minimally important difference for the EQ-5D Summary Index. Also, we observed that physicians were significantly more likely to be satisfied with PARPi treatment over chemotherapy, at least partly reflecting the improved quality of life reported with PARPi.

Several limitations of the study are noted. Data were more likely to be collected on patients who visit their physician more frequently, and the data relied on the accurate reporting of the physician and the patient. Also, patient-reported forms were voluntary, and patients with more symptom burden may have been less likely to participate. On the other hand, it is possible that patients with better general health visit the clinic less often and may also be less likely to participate. In addition, baseline PRO questionnaires were not collected, precluding longitudinal analyses of PROs. The sample sizes for both treatment groups were relatively small, limiting the statistical power of the analyses. In addition, adjustments were made among observed known differences between the 2 cohorts. Although the eligibility criteria involved all adult patients with g*BRCA1/2*mut HER2– ABC, only female patients completed the questionnaires. Also, apart from capecitabine, chemotherapies are administered intravenously, whereas PARPi are administered orally. The different administration routes between chemotherapy and PARPi therapy may influence patients QoL measures. We also note that we did not capture data on whether PARPi-treated patients had received antiemetic premedication, which could have affected the QoL measures. Moreover, it cannot be excluded that patient knowledge that a targeted therapy based on a reliable predictor is available can positively influence PROs.

## Conclusions

In this real-world, multinational population of patients with g*BRCA1/2*mut HER2− ABC, those receiving PARPi therapy reported significantly better QoL scores in multiple dimensions on the EORTC QLQ-C30, QLQ-BR23, and EQ-5D-5L questionnaires compared with patients receiving chemotherapy. On only 1 index, the EORTC QLQ-C30 nausea/vomiting scale, did patients receiving chemotherapy report a significantly better score than those receiving PARPi therapy. This is potentially due to olaparib therapy, which was associated with worse PROs for nausea and vomiting among patients with g*BRCA1/2*mut HER2− ABC in the OlympiAD trial. These findings in this real-world population are consistent with the improved PROs observed in the EMBRACA and OlympiAD trials and further support the use of PARPi in this patient population.

## Supplementary Information

**Additional file 1: Supplementary Table S1.** Comparison of demographic and clinical data between study populations.

## Data Availability

Data collection was undertaken by Adelphi Real World as part of an Adelphi Disease Specific Programmes™ independent survey subscribed by multiple pharmaceutical companies, one of which was Pfizer Inc. Pfizer did not influence the original survey through either contribution to the design of questionnaires or data collection. The survey described here using data from the Adelphi Disease Specific Programmes™ was funded by Pfizer. Publication of study results was not contingent on the subscriber’s approval or censorship of the manuscript. All data that support the findings of this study are the intellectual property of Adelphi Real World. All requests for access should be addressed directly to Katie Lewis at katie.lewis@adelphigroup.com.

## Abbreviations

ABC: Advanced breast cancer
g*BRCA1/2*mut: Germline breast cancer susceptibility gene 1 or 2 mutation
CTSQ: Cancer Therapy Satisfaction Questionnaire
GHS: Global health status
HER2–: Human epidermal growth factor receptor 2–negative
ECOG: Eastern Cooperative Oncology Group
PARPi: Poly (ADP-ribose) polymerase inhibitors
EORTC: European Organization for Research and Treatment of Cancer
EQ-5D-5L: EuroQol 5-Dimensions 5-Level
IPWRA: Inverse-probability−weighted regression adjustment
PFS: Progression-free survival
PRF: Patient record form
PRO: Patient-reported outcomes
QLQ-BR23: Quality-of-life questionnaire breast cancer–specific module
QLQ-C30: Quality-of-life questionnaire core 30 module
QoL: Quality of life
VAS: Visual analog scale
