# Supplementary material for: Real-world patient-reported outcomes and physician satisfaction with poly (ADP-ribose) polymerase inhibitors versus chemotherapy in patients with germline BRCA1/2-mutated human epidermal growth factor receptor 2–negative advanced breast cancer from the United States, Europe, and Israel
Source: BMC Cancer. 2022 Dec 22;22:1343. doi: 10.1186/s12885-022-10325-9 (PMC9773591; doi:10.1186/s12885-022-10325-9)
Supplement: Supplementary file 1 — Additional file 1: Supplementary Table S1. Comparison of demographic and clinical data between study populations. [file 12885_2022_10325_MOESM1_ESM.pdf]

Supplementary Table S1. **Comparison of the Demographic and Clinical Characteristics Between the Germline *BRCA1/2* Mutated Population With Missing Data and the IPWRA-Adjusted Germline *BRCA1/2* Mutated Population With Complete Patient-Reported Data Available**

	Overall Population (n=562)	Missing Data Population (n=466)	IPWRA Population (n=96)	P Value
Age[a]				
n	514	418	96	0.006[b]
Mean (SD)	54.2 (12.7)	55.0 (12.8)	51.1 (11.5)	
Median (IQR)	55.2 (44.3, 64.8)	56.8 (45.3, 65.2)	49.3 (41.0, 60.5)	
Charlson Comorbidity Index				
n	562	466	96	0.144[b]
Mean (SD)	7.3 (2.3)	7.4 (2.2)	7.0 (2.6)	
Median (IQR)	8.0 (8.0, 8.0)	8.0 (8.0, 8.0)	8.0 (8.0, 8.0)	
Hormone receptor status, n (%)				
n	543	447	96	<0.001[c]
TNBC	162 (29.8)	99 (22.1)	63 (65.6)	
HR+/HER–	381 (70.2)	348 (77.9)	33 (34.4)	
ECOG performance status,[a] n (%)				
n	562	466	96	<0.001[d]
0	142 (25.3)	101 (21.7)	41 (42.7)	
1	310 (55.2)	268 (57.5)	42 (43.8)	
2	85 (15.1)	73 (15.7)	12 (12.5)	
3	14 (2.5)	14 (3.0)	0 (0.0)	
4	11 (2.0)	10 (2.1)	1 (1.0)	
Stage, n (%)				
n	559	463	96	0.217[c]
IIIa/IIIb	87 (15.6)	68 (14.7)	19 (19.8)	
IV	472 (84.4)	395 (85.3)	77 (80.2)	
Lines of therapy for ABC				
n	560	464	96	0.511[b]

Mean (SD)	1.6 (0.7)	1.6 (0.6)	1.6 (0.7)	
Median (IQR)	1.0 (1.0, 2.0)	1.0 (1.0, 2.0)	1.0 (1.0, 2.0)	
Baseline toxicity, n (%)				
n	562	466	96	
Constitutional	194 (34.5)	155 (33.3)	39 (40.6)	0.195[c]
Pulmonary	112 (19.9)	89 (19.1)	23 (24.0)	0.326[c]
Gastrointestinal	169 (30.1)	150 (32.2)	19 (19.8)	0.020[c]
Dermatologic	93 (16.5)	74 (15.9)	19 (19.8)	0.366[c]
Neurologic	47 (8.4)	39 (8.4)	8 (8.3)	1.0[c]
Psychological	79 (14.1)	69 (14.8)	10 (10.4)	0.333[c]
Physical	140 (24.9)	99 (21.2)	41 (42.7)	<0.001[c]
Pain	179 (31.9)	154 (33.0)	25 (26.0)	0.190[c]

ABC=advanced breast cancer; *BRCA1/2*=breast cancer susceptibility gene 1 or 2; ECOG, Eastern Cooperative Oncology Group; HER2–, human epidermal growth factor receptor 2–negative; HR+, hormone receptor–positive; IQR=inter quartile range; TNBC=triple-negative breast cancer.

[a]At time of therapy initiation.

[b]Student's *t* test.

[c]Fisher's exact test.

[d]Mann-Whitney U test.
